# Baduanjin exercise combined with moxibustion box for gastrointestinal dysfunction in the elderly: a protocol of prospective, randomized controlled trial

**DOI:** 10.3389/fmed.2025.1620346

**Published:** 2025-07-28

**Authors:** Jing Zhu, Dongxia Zhang, Lu Yang, Hong Liang, Qingyan Liu, Jinyan Liang, Zunjiang Li, Banghan Ding, Danwen Zheng

**Affiliations:** ^1^The Second Affiliated Hospital of Guangzhou University of Chinese Medicine, Guangzhou, China; ^2^The Second Clinical College of Guangzhou University of Chinese Medicine, Guangzhou, China

**Keywords:** Baduanjin exercise, moxibustion box, gastrointestinal dysfunction, elderly patients, protocol, rct

## Abstract

**Objective:**

To synthesize and compare the results of a controlled trial of traditional Chinese medicine (TCM) nursing techniques, Baduanjin exercise (BDJE) combined with moxibustion box (MB) intervention measures, to improve gastrointestinal dysfunction (GID) in the elderly.

**Design:**

A prospective randomized controlled trial.

**Method:**

Participants were recruited from the Guangdong Provincial Hospital of Traditional Chinese Medicine and randomly assigned to either the intervention group or the conventional treatment group using a random number table generated by SAS software. The intervention measures included the use of BDJE plus MB for 2 weeks. The main outcome measures were the Gastrointestinal Symptom Rating Scale (GSRS) and clinical efficacy. Secondary outcome measures included bowel sounds, changes in abdominal circumference, frequency of bowel movements per day, and stool morphology. Measure the outcome before and 2 weeks after treatment.

**Discussion:**

Gastrointestinal dysfunction (GID) has a high incidence rate in severely ill patients and is associated with poor prognosis. Elderly patients with GID are affected by this disease, which reduces their quality of life and, subsequently, affects their mental health. The treatment of elderly patients with GID includes Western drug treatment (WDT) and traditional Chinese medicine. However, the therapeutic effect of WDT during enteral nutrition in critically ill patients is unsatisfactory. BDJE combined with MB in TCM nursing technology has the characteristics and advantages of simple operation, low price, convenience, and effectiveness, and is more easily accepted by patients. Therefore, this study aimed to compare the clinical efficacy of WDT and the combination of BDJE and MB in elderly patients with GID.

**Impact:**

This study provides evidence of the clinical efficacy of BDJE combined with MB in treating elderly patients with GID.

**Trial registration:**

http://itmctr.ccebtcm.org.cn/, Identifier, ITMCTR2025000167.

## 1 Introduction

Gastrointestinal dysfunction (GID) broadly refers to functional impairment of the GI tract that may include disturbances in motility and/or absorption, breaches in mucosal integrity, changes in the microbiome, increased intra-abdominal pressure, impaired mesenteric perfusion infections of the GI tract, and other clinical consequences (1). Studies have shown that the incidence of GID in patients admitted to the ICU in Europe and the United States is 30–70% (2). Additionally, it has been demonstrated that only 20% of patients receive a diagnosis of gastrointestinal dysfunction prior to being sent to the intensive care unit, and that the frequency rises to 82% after a week of admission (3). The main symptoms of GID include decreased appetite, constipation, diarrhea, and weakened bowel sounds in elderly adults with gastrointestinal motility and coordination dysfunction brought on by aging, as well as decreased gastric tone, intestinal contractility, rectal pressure sensation, and various digestive enzymes, among other things (4–6). The everyday activities of elderly individuals with GID are negatively impacted, their yearly income declines, their mental health deteriorates, and the healthcare system bears a significant financial burden (7, 8), there is an urgent need to focus on alleviating the symptoms and negative impacts of GID faced by elderly patients.

Traditional Chinese medicine (TCM) and Western drug therapy (WDT) are used to treat GID in elderly Chinese patients. In critically ill patients, intolerance to WDT is common during enteral feeding and is linked to the clinical manifestations of malnutrition. Delayed gastric emptying is one of the primary causes of feeding resistance for gastrointestinal reasons (such as big gastric leftovers, bloating, vomiting, or subjective discomfort), and drugs can improve tolerance in critically ill patients by promoting gastric emptying (9). The benefits of TCM therapies, such as their ability to reduce side effects and dredge meridians and collaterals, have made them popular among elderly patients with GID (10, 11). There are two types of TCM: internal and external. External treatments include acupuncture, moxibustion boxes, acupoint plastering, acupoint embedding, Baduanjin exercise (BDJE), and others (12). Internal treatments primarily consist of decoction and traditional Chinese patent medications (13). As a crucial component of TCM’s clinical diagnostic and treatment system, external treatment upholds the benefits of TCM, including its ease of use, affordability, practicality, and efficacy, and it has demonstrated significant vitality in the clinic (14). Furthermore, there are several benefits to using TCM externally, including increased safety, fewer side effects, improved clinical efficacy, and improved clinical symptoms for patients (15, 16).

Baduanjin exercise (BDJE), an external, non-pharmacological TCM treatment with over 1,600 years of history in China, is a model of the combination of movement and static in ancient Chinese guiding techniques that can achieve the goal of maintaining fitness and health by dredging the triple energizer, regulating qi and blood, and harmonizing yin and yang (17). BDJE increases mood, modifies intestinal and brain peptides, lowers the recurrence rate, and lessens clinical symptoms in patients with irritable bowel syndrome and constipation (18). According to a study, in patients with functional dyspepsia, BDJE significantly reduces epigastric discomfort, epigastric distension, belching, early satiety, nausea, and decreased appetite. It particularly improves decreased appetite, which has a more substantial therapeutic effect (19). The elderly can easily learn how to use the BDJE, and it is easy to move, breathe naturally, control, and operate. Nonetheless, the broader population continues to accept this. The foundation of MB is the idea of meridian science, which works by stimulating certain acupuncture points with light and warmth (20, 21). Patients are not harmed by the comparatively low heat of MB. Their level of acceptance is higher and they feel more at ease (22). By raising the surface temperature of the patient’s abdomen, MB can help lessen the gastrointestinal tract’s smooth muscle spasms, encourage the gastrointestinal tract’s peristalsis, improve the surrounding tissues’ blood circulation symptoms, and improve the patient’s gastrointestinal function (23). A trial has shown that MB can improve the symptoms of belching, fatigue, gastric distension, and gastric mucosal inflammation, and improve the quality of life, with a short time of clinical symptomatic relief and a low recurrence rate (24). It can also promote intestinal peristalsis and improve intestinal obstruction (25). BDJE and MB are two TCM nursing-guided techniques conducted by nurses in clinics, and the combination of these techniques seems to exert better efficacy for elderly patients with GID by promoting blood circulation, regulating immunity, reducing gastrointestinal spasms, improving gastrointestinal function in the elderly, and enhancing their quality of life (26).

However, due to the lack of sufficient research, the efficacy of BDJE combined with MB in the treatment of elderly GID has yet to be clearly elucidated. Thus, in this study, we aim to explore and demonstrate the efficacy and safety of BDJE combined with MB in the treatment of elderly patients with GID.

## 2 Methods

### 2.1 Trial design and ethical approval

This study is a prospective, randomized controlled clinical trial that has been approved by the Ethics Committee of Guangdong Provincial Hospital of Traditional Chinese Medicine with a follow-up period of 2 weeks from January 1, 2025, to December 31, 2026 (Ethics approval No: ZF2024-430-01). All recruited patients have understood and signed an informed consent form before participating in the trial. The study is registered at http://itmctr.ccebtcm.org.cn/ (ITMCTR2025000167).

### 2.2 Participants selection

#### 2.2.1 Inclusion criteria

Participants are included according to the following criteria: ① All patients meet the diagnosis of GID in Multiple Organ Dysfunction Syndrome; ② Patients age 60 years and above; ③ Patients do not participate in other clinical research; ④ Patients voluntarily participate in this clinical trial, understand and sign the informed consent form; ⑤ Patients have not participated in any other clinical trials in the past 3 months.

#### 2.2.2 Exclusion criteria

Participants are excluded if they meet the following criteria: ① Patients who cannot cooperate with BDJE practice; ② Patients allergic to moxa stick components or intolerant to MB; ③ GID caused by trauma or other reasons; ④ Patients with malignant tumors, severe liver/kidney dysfunction, or hematological disorders; ⑤ Patients with prior gastrointestinal diseases (e.g., gastrointestinal surgery, irritable bowel syndrome, inflammatory bowel diseases, or malignancies).

#### 2.2.3 Diagnostic criteria of GID in multiple organ dysfunction syndrome

The study uses the “Multiple Organ Dysfunction Syndrome (Disease Staging Diagnosis and Severity Scoring Criteria)” discussed and approved by the National Emergency Medicine Academic Association for Multiple Organ Dysfunction Syndrome (Lushan Conference) as the diagnostic criteria (27). Gastrointestinal functional disorders are present in patients with multiple organ dysfunction syndrome. No abdominal bloating or normal bowel sounds are scored as 0 points. Abdominal bloating and decreased bowel sounds are scored as one point. High abdominal bloating and almost complete disappearance of bowel sounds are scored as 2 points. Paralytic intestinal obstruction and stress-induced bleeding (those who meet either criterion are diagnosed) are scored as 3 points. A severity score of ≥1 and <3 indicates gastrointestinal dysfunction, whereas a score of 3 indicates gastrointestinal failure.

#### 2.2.4 Screening process

All elderly patients diagnosed with GID who meet the inclusion criteria are recruited from the Emergency Department of Guangdong Provincial Hospital of Traditional Chinese Medicine. Any participant who meets the exclusion criteria is removed by the researchers, and the reasons are recorded. Subsequently, the recruited patients are randomly divided into two groups (BDJE+MB group and control group) according to the random table numbers obtained using SAS software.

#### 2.2.5 Exit or suspend criteria

The experiment is carried out to guarantee sufficient statistical efficiency and provide significant research findings once the recruitment sample size has been established prior to the investigation and achieves the predetermined level. The researcher must immediately cease ensuring the participants’ safety and rights if significant safety or ethical concerns arise during the experiment. At the same time, the trial is terminated if subjects decide to withdraw for personal reasons. Patients who meet the inclusion criteria but do not finish the trial are deemed to have dropped out or discontinued therapy. This includes: ① Errant cases that do not meet the inclusion criteria; ② patients who experience worsening of their condition and require emergency medical intervention; ③ poor patient compliance, voluntary withdrawal from the study, or incomplete adherence to the study protocol (less than 60%, the 60% compliance threshold aligns with WHO adherence guidelines for elderly trials (28) and prior TCM studies (29)); ④ those with incomplete data affecting efficacy rating and safety evaluation.

### 2.3 Sample size calculation

The sample size was determined based on the minimal clinically important difference (MCID) of the primary endpoint, the Gastrointestinal Symptom Rating Scale (GSRS) total score. Drawing from recent studies on MCID methodologies for gastrointestinal scales, the established MCID for the 15-item GSRS in elderly populations is 7.86 points (95% CI, 7.86–9.29) when calculated using the 1.96 standard error of measurement (SEM) method, as recommended for distribution-based approaches (30). We conservatively set the expected between-group difference at 8 points (exceeding this MCID threshold) with an estimated standard deviation (SD) of 16.4 points (31), derived from a 2023 multicenter study of elderly diabetic patients with gastrointestinal symptoms reporting GSRS-related SDs of 16.36 ± 3.70 (30). Using a two-sided *α* = 0.05 and *β* = 0.1 (90% power) (32), the initial calculation indicated 199 participants per group. To account for potential attrition, we referenced contemporary geriatric trial data reporting 8.8% dropout rates in optimized multisite studies (31), and adopted a conservative 10% attrition rate given our trial’s centralized monitoring system. Thus, the final adjusted sample size is 222 per group (199 / 0.9), rounded to 200 per group (400 total) to maintain balanced allocation while exceeding minimum requirements.

### 2.4 Interventions and comparison

The control group receives standardized Western Drug Treatment (WDT) based on the current Chinese expert consensus guidelines for managing gastrointestinal dysfunction in the elderly (33–36). The treatment protocol follows a symptom-driven algorithm to ensure scientific rigor and replicability. Permissible medications include prokinetic agents such as compound azithromycin enteric-coated tablets and mosapride to alleviate gastrointestinal motility disorders manifested by symptoms like bloating, early satiety, and delayed gastric emptying; digestive enzyme preparations (various compound digestive enzyme formulations) to improve symptoms related to decreased digestive function, such as loss of appetite and dyspepsia; acid-suppressive drugs including proton pump inhibitors and H2 receptor antagonists for patients with hyperacidity or gastric mucosal injury; and mucosal protectants such as montmorillonite powder and sucralfate suspension to protect the gastrointestinal mucosa. Treatment is administered according to an algorithmic decision-making process, selecting specific drugs or combinations based on the predominant clinical presentation and signs. Dosage and duration conform to expert consensus recommendations. Adverse effects are closely monitored, and treatment adjustments are made only in response to significant adverse events. The use of traditional Chinese medicine, acupuncture, or other non-Western therapies is strictly prohibited in the control group. This standardized WDT regimen ensures consistency and scientific validity of treatment, providing a reliable baseline for comparison of clinical outcomes.

**Figure 1:**
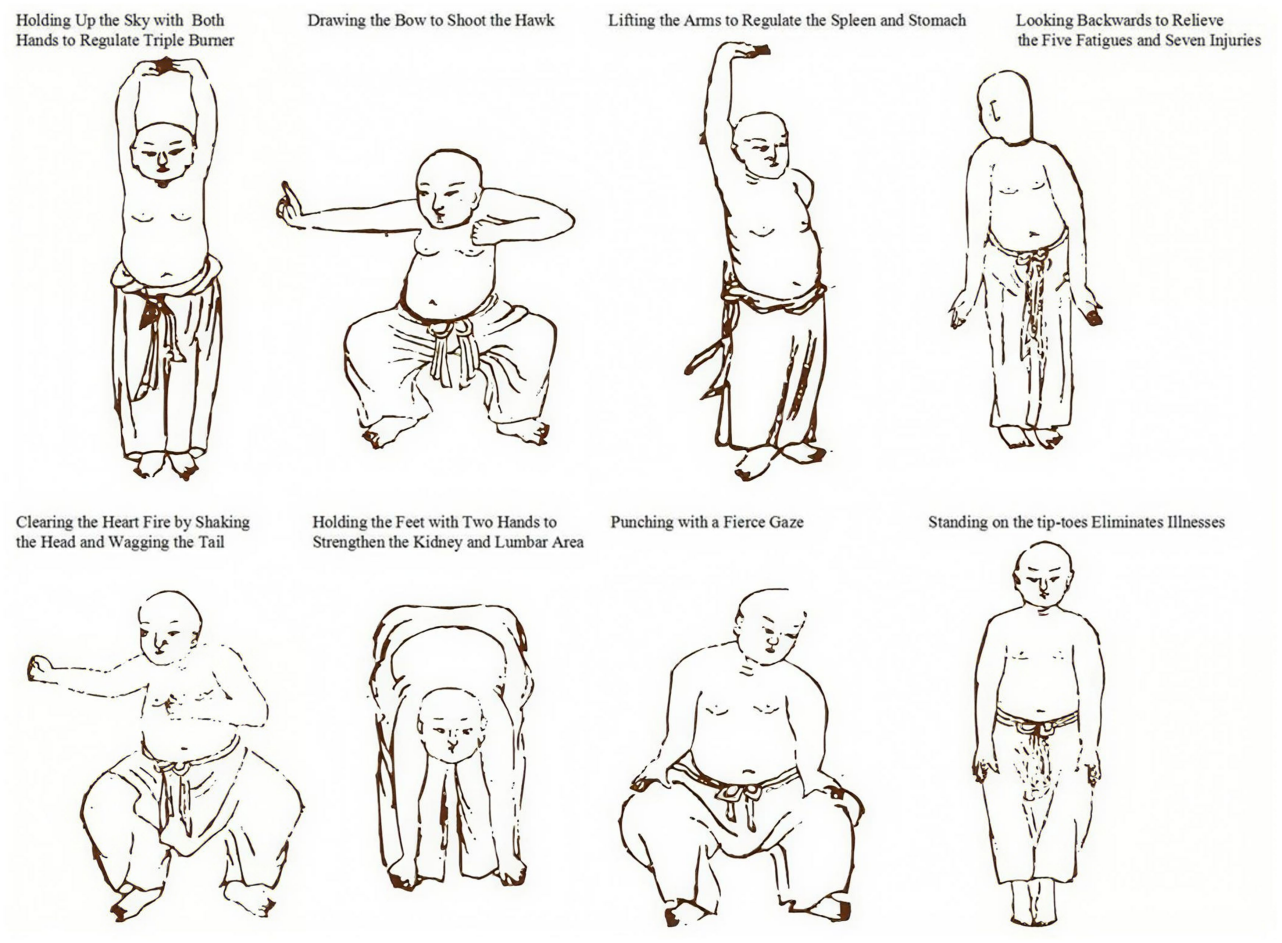
The diagram of Baduanjin exercise.

On the basis of the control group, the experimental group is treated with BDJE combined with MB. The patients practice a set of BDJE guidance techniques half an hour after breakfast every morning (referring to the BDJE fitness exercise method issued by the General Administration of Sport of China in 2003) and should avoid being too full before practice (Figure 1). The duration of each practice session is controlled within 30 min. After practicing BDJE for 20 min, MB treatment is performed (following the “TCM Nursing Routine Technical Operation Specification” of the State Administration of TCM). The elderly patients lie supine on the bed, and the nurses take three moxa sticks, ignite them, place them in moxa boxes in different zones, and position the boxes flat on the abdomen with the Shenque acupoint at the center. The moxibustion treatment lasts for 20 min. To ensure the safety of moxibustion, a cotton cloth barrier is placed between the moxibustion box and skin. Treatment is stopped immediately for any redness, pain, or discomfort, and documented as an adverse event. After moxibustion, elderly patients drink 50 mL of diluted saline water. The two treatment courses last 14 days, with 7 days as one course and 2 days of rest. After two courses of treatment, the efficacy is observed and compared.

#### 2.4.1 Standardization of interventions

The Baduanjin exercise (BDJE) protocol was administered by nurses certified through the China Health Qigong Association (minimum 50-h training program). Participants followed the official 2003 instructional video during supervised sessions to ensure movement consistency. Adherence was quantified via attendance logs and triaxial accelerometers (ActiGraph GT9X, sampling rate 30 Hz) to monitor exercise intensity. For moxibustion box therapy, licensed Traditional Chinese Medicine (TCM) nurses with ≥3 years of clinical experience performed the intervention using standardized moxa sticks (Nanyang Aiye Co.; dimensions: 25 mm diameter × 200 mm length) ignited for 5 min prior to application. Thermal output was regulated in real-time using infrared imaging (FLIR E6 thermal camera) to maintain abdominal surface temperature at 43 ± 2°C throughout the 20-min treatment.

### 2.5 Primary outcome measurement

The primary outcome measures include the Gastrointestinal Symptom Rating Scale (GSRS) and clinical efficacy. The GSRS is used to evaluate the levels of gastrointestinal symptoms before and after treatment. The GSRS is a disease-specific tool consisting of 15 items organized into five symptom clusters that correspond to various gastrointestinal complaints. Constipation, reflux, indigestion, diarrhea, and abdominal discomfort are represented by the five symptom clusters. A seven-grade Likert-type scale, with 1 denoting no troublesome symptoms and 7 denoting extremely bothersome symptoms, is used in the GSRS. The validity and reliability of the GSRS have been thoroughly documented, and norm values for the general public are available (37, 38). The clinical efficacy evaluation criteria include: ① Significant effect: improvement in the severity of GID by two or more levels; ② Effective: improvement of GID severity by one level; ③ Ineffective: patients with GID whose severity improvement is less than level 1; ④ Serious: defined as either ≥3-point increase in total GSRS score, or progression to gastrointestinal failure (MODS score = 3).

### 2.6 Secondary outcome measurement

In addition to demographic data and general vital signs between the two groups, the secondary outcome measurements include: ① bowel sounds; ② changes in abdominal circumference. The abdominal circumference is measured with a tape measure around the abdomen via the navel. ③ Frequency of bowel movements per day and stool morphology (Bristol Stool Form Scale, BSFS). Both the treatment and control groups are interviewed, and their stool characteristics are recorded on the day before treatment and during the second weekend of the experiment.

#### 2.6.1 Bristol stool form scale

**Figure 2:**
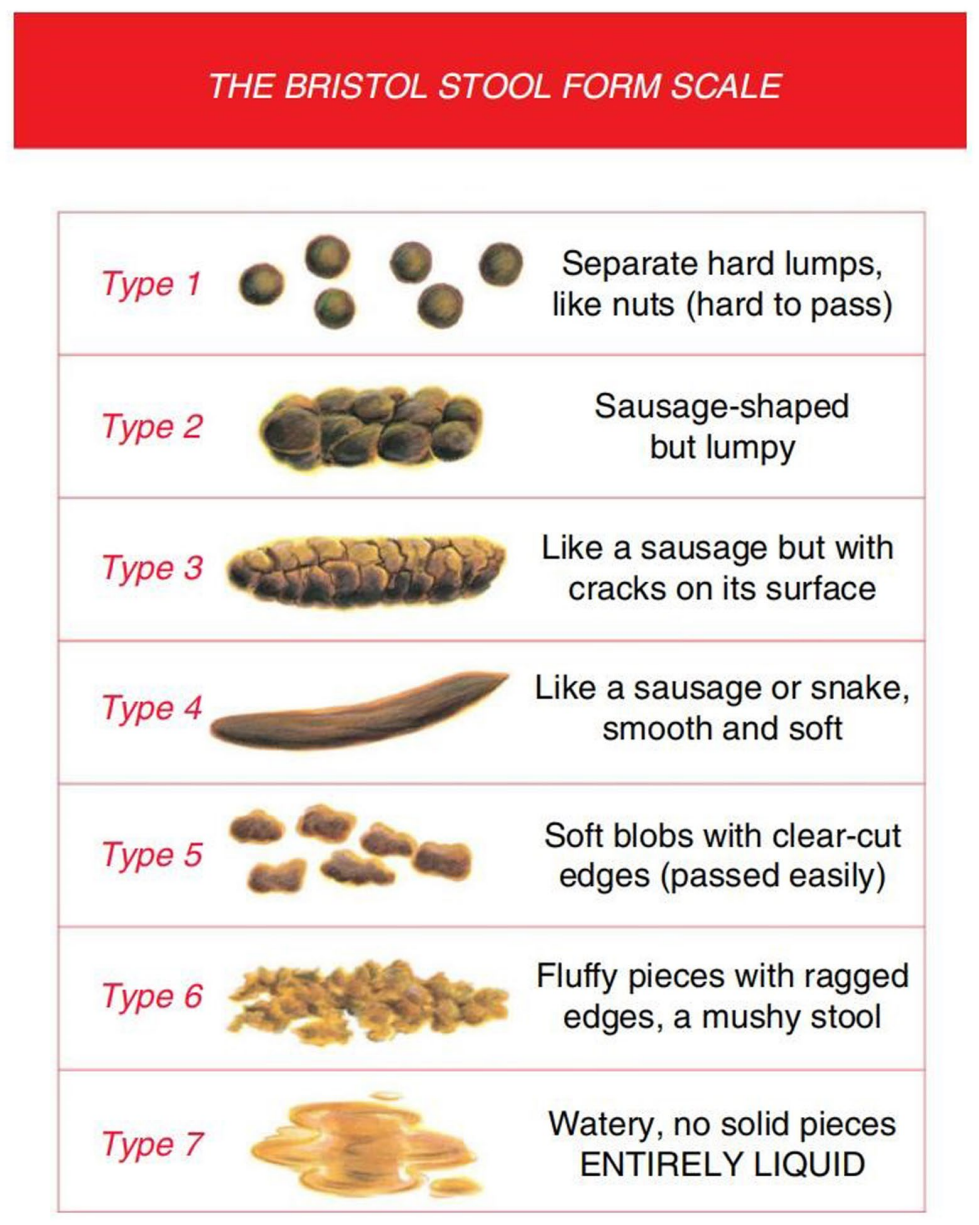
The Bristol Stool Form Scale.

The Bristol Stool Form Scale (BSFS) is a 7-point scale used extensively in clinical practice and research for stool form measurement, which has undergone limited validity and reliability testing (39). The use of this tool is conducted under appropriate copyright permissions, and all data collection and analysis adhere to its requirements. The BSFS can also detect clinically significant differences in measurement structures (structural validity). For example, fecal consistency and fecal moisture are negatively correlated with gut transit time (GTT), and there is ample evidence to suggest that fecal types measured using the BSFS are negatively correlated with all or part of the GTT in healthy adults (40). The BSFS is an ordinal scale of stool types, ranging from the hardest (Type 1) to the softest (Type 7). According to BSFS: Type 1: separated hard lumps; Type 2: clumps; Type 3: dry and cracked sausages; Type 4: soft sausages; Type 5: soft lumps; Type 6: mud-like; Type 7: watery stool (41). Types 3, 4, and 5 are generally considered the most “normal” forms of feces (31) and are typical forms in cross-sectional surveys of healthy adults (42), while the rest are abnormal. For greater clarity, graphic representations of each stool type are incorporated, even though the original development only used written descriptors (Figure 2).

### 2.7 Quality control

**Figure 3:**
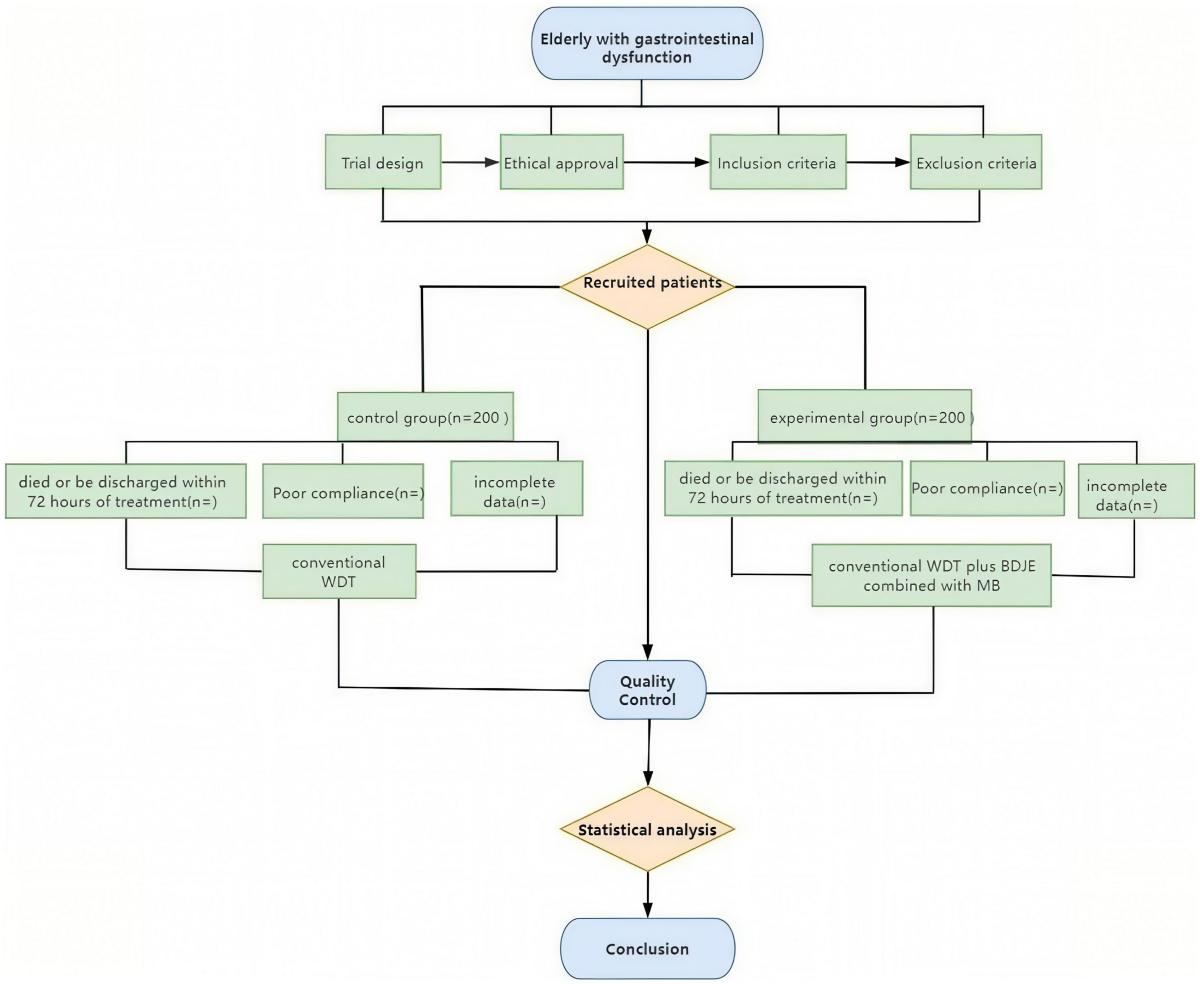
The flow chart of the clinical procedures through the trail.

Clinical researchers undergo specialized clinical training to coordinate the research progress prior to the trial’s formal start. We focus on standard operating procedures and the project’s execution strategy to improve the reliability of findings, observer consistency, and internal observation uniformity. Each clinical researcher learns about the research procedures and implementation details. To reduce patient withdrawal rates, we encourage patients to adhere strictly to the treatment plan throughout the study to maintain compliance. Participants who withdraw are asked to explain their reasons, and their responses are reviewed. Only researchers involved in the study may access confidential medical records, and they must sign the investigator’s declaration. The ethics committee reviews all clinical study documentation. Subject-specific identifying information is excluded, and anonymized data processing is employed. Outcome assessors collecting GSRS scores, bowel sounds, abdominal circumference, and stool characteristics will be blinded to group allocation. They operate from a dedicated assessment center physically separated from the intervention units, receiving only coded identifiers (e.g., ‘GID-001’). Unblinding will occur exclusively after database lock and prior to final statistical analysis. Data analysts will receive anonymized datasets labeled ‘Group A/B’ without intervention details. Due to the nature of the interventions (BDJE and MB), participants and nursing staff administering treatments cannot be blinded. The clinical procedures are depicted in Figure 3.

### 2.8 Data analysis method

The data is processed using SPSS software (version 21.0), and the mean ± standard deviation is utilized to express measured data. Statistical analysis follows the intention-to-treat (ITT) principle. Missing data are handled by multiple imputation (5 imputed datasets) using the SPSS Multiple Imputation (MVA) module. Sensitivity analysis with per-protocol population will be performed as supplementary validation. Quantitative data that fit the normal distribution and have equal variance are analyzed using the t-test; intra-group comparisons before and after are analyzed with the paired t-test; inter-group comparisons are analyzed with the independent sample t-test; categorical data are counted using the chi-square test; and rank data with two independent samples of ordered variables are compared using the rank-sum test.

### 2.9 Safety monitoring

A stratified safety protocol was implemented to address age-specific risks. For Baduanjin exercise, pre-session screening using the Borg CR-10 Scale mandated termination if perceived exertion exceeded ≥6 (“very hard” intensity), with absolute contraindications including unstable angina and acute vertigo. During moxibustion therapy, continuous infrared thermography (FLIR E6) triggered immediate cessation if skin temperature surpassed 47°C, the epidermal damage threshold established by ISO 13732-1:2006. All adverse events (AEs) were documented per CONSORT Extension for TCM guidelines, including severity grading (CTCAE v5.0) and causality assessment. An independent Data and Safety Monitoring Board (DSMB) comprising a geriatrician, TCM specialist, and biostatistician conducted monthly unblinded safety reviews to evaluate AE profiles and recommend protocol modifications.

## 3 Discussion

GID is characterized by damage to the intestinal parenchyma or function, leading to impaired digestion, nutrient absorption, and/or mucosal barrier function (9). One study showed that the incidence rate of gastrointestinal dysfunction in severe patients was 68.08%, which seriously affected the prognosis of patients (43, 44). According to current research, the pathogenesis of GID can be attributed to the following six aspects (45): first, defects in anatomical tissues caused by gastrointestinal surgery or non-gastrointestinal surgery; second, gastrointestinal digestion and absorption dysfunction; third, impaired intestinal mucosal function leading to intestinal barrier dysfunction (46); and fourth, gastrointestinal motility disorders caused by taking drugs that affect the gut microbiota (47). The fifth is related to the low levels of citrulline and glutamine in the human body (48). The sixth factor is the impact of emotional changes and disorders, which aligns with emerging evidence on gut-brain axis dysregulation in GID pathogenesis (49, 50). Recent advances in GID management highlight gut-brain axis modulation (e.g., vagus nerve stimulation) and microbiome-targeted therapies (51, 52). A study confirms that 54–73% of functional GID patients exhibit psychological comorbidities (53), with dysbiosis exacerbating intestinal barrier damage through inflammatory cascades (54). Current microbiome interventions include probiotics (e.g., *Lactobacillus rhamnosus* GG improving gastric motility) and rifaximin (55, 56), while neuromodulation strategies target emotional comorbidities (57).

Currently, conventional WDT mainly focuses on sufficient capacity supply, promotes gastrointestinal motility, assists in defecation, maintains intestinal microbiota balance, and protects the gastrointestinal mucosa (58). A study has shown that conservative fluid resuscitation and active enteral feeding can improve gastrointestinal function in mildly ill patients, but to some extent exacerbates gastrointestinal dysfunction in critically ill patients (59). Compared with WDT, TCM has diverse methods for treating gastrointestinal dysfunction, with stable efficacy and high safety.

In recent years, many clinical studies have found that BDJE can regulate gut microbiota, promote intestinal health, and improve the negative emotional and psychological states of patients with GID (60, 61). MB can compensate for the shortcomings of acupuncture and medicine, with a large moxibustion area, mild heat, and high comfort, making it easy for patients to accept (62). Therefore, the combination of BDJE and MB can promote the recovery of gastrointestinal function, regulate immunity, reduce gastrointestinal spasms, improve the quality of life of elderly patients with GID, and restore daily social activities as much as possible. Our BDJE+MB approach aligns with these trends by combining neuromodulation (exercise-induced microbiota optimization) (63) and local anti-inflammatory effects (moxibustion-mediated barrier repair) (12). Unlike pharmacotherapies, this non-invasive strategy may benefit elderly patients with polypharmacy risks, particularly given age-related increases in intestinal permeability and systemic inflammation.

Many studies have evaluated the intervention measures of using acupuncture and other TCM external treatment methods in the community or hospital to treat elderly patients with GID and have determined positive effects (64, 65). However, no research has tested the effectiveness of using RCTs to treat elderly patients with GID in a hospital setting using a combination of BDJE and MB. Therefore, this study aims to address this gap by evaluating the effectiveness of BDJE+MB in a hospital environment. The findings may provide novel strategies to reduce adverse outcomes, enhance clinical efficacy, shorten hospitalization, lower costs, and improve quality of life for elderly GID patients.

### 3.1 Limitations

This trial has several limitations: ①Blinding constraints: Participants and nursing staff cannot be blinded due to the visible nature of BDJE and MB, risking performance bias. ② Placebo effect: The experimental group receives more contact time (BDJE + MB), potentially amplifying placebo effects. ③ Intervention standardization: Variability in BDJE execution may occur despite standardized training. To mitigate this, all nurses will complete a certified 50-h training program and follow video demonstrations during sessions. ④ Generalizability: Results may not extend to severe GID cases (MODS score ≥3) or non-geriatric populations.
